# Clinical Confidence in Endodontics: A Cross-Sectional Study of Undergraduate Students’ First Patient Experience

**DOI:** 10.4317/jced.63542

**Published:** 2026-02-26

**Authors:** Isabel Gálvez, Ana Antoranz, Natalia Navarrete, Marta Muñoz-Corcuera

**Affiliations:** 1 Universidad Europea de Madrid. School for Doctoral Studies and Research; 2 Universidad Europea de Madrid. Faculty of Biomedical and Health Sciences. Department of Clinical Dentistry-Pregraduate studies

## Abstract

**Background:**

Endodontics is a complex and often stressful dental procedure for undergraduate students, requiring the development of fine motor skills and manual dexterity. Performing this treatment for the first time on patients can be particularly challenging. This cross-sectional observational study aims to evaluate the perceptions and self-reported confidence levels of fifth-year dental students when performing their first endodontic treatment on patients.

**Material and Methods:**

Following their first root canal treatment performed in the clinical setting under the supervision of an endodontics instructor, fifth-year students completed a structured questionnaire. The survey included Likert-scale items assessing perceived case difficulty and confidence across various stages of the procedure. Additionally, an open-ended question was included to gather qualitative feedback for potential improvements in endodontic education.

**Results:**

A total of 179 students completed the questionnaire, yielding a 89.5% response rate. Molars were reported as the most challenging teeth to treat, while premolars were the most frequently treated. Students identified root canal obturation as the most complex phase of the procedure and reported the lowest confidence during working length determination.

**Conclusions:**

Enhancing the number of preclinical workshops prior to patient-based endodontic treatments may improve students' confidence and help them develop the manual skills necessary for successful clinical performance.

## Introduction

Endodontic treatment is a core clinical requirement for fifth-year undergraduate dental students, forming part of their clinical internship with adult patients. To successfully complete this requirement, students must be capable of establishing an accurate diagnosis, understanding the available treatment options, and competently performing each step of the endodontic procedure (1). In recent years, advancements in endodontic materials and techniques have been integrated into dental education, enhancing the learning experience and clinical preparedness of undergraduate students (2). Despite these improvements, endodontic procedures remain among the most technically demanding and stressful aspects of dental training. Contributing factors include limited direct vision, the need for refined tactile skills, complex root canal anatomy, and the potential for complications during various stages of treatment-such as access cavity preparation, canal location, working length determination, instrumentation, irrigation, and obturation-often compounded by students' low confidence levels (2 - 4). The learning curve in endodontics is steep, requiring students to translate theoretical knowledge into clinical practice. Initially, manual skills are developed through simulation exercises using artificial teeth, where students follow standardized protocols with specific instruments and materials. However, when transitioning to real patient care, students frequently experience anxiety and insecurity due to limited autonomy, necessitating close supervision by instructors to ensure proper treatment execution (5 , 6). Understanding students' perceptions of endodontic procedures is essential for evaluating the effectiveness of current teaching methods and identifying areas for improvement. Student feedback offers valuable insights into the challenges they face and highlights the aspects they prioritize in clinical practice, thereby informing curriculum development and instructional strategies (7). Previous studies have consistently identified canal location and obturation as the most challenging and anxiety-inducing steps for students during their initial clinical experiences (4 , 8). The literature also emphasizes the technical difficulties associated with root canal localization and working length (WL) determination. Canal location demands a thorough understanding of internal anatomy and precise tactile control, while accurate WL determination is critical to avoid procedural errors and prevent apical pathology (9 - 11). These challenges are further compounded during obturation, a technically sensitive phase requiring a three-dimensional seal to prevent reinfection (4 , 8). Based on these findings, this study was designed with the expectation that fifth-year dental students would perceive root canal location, working length determination, and canal obturation as the most challenging phases of endodontic treatment during their first clinical experience. This triad reflects both the technical complexity of these procedures and the findings of previous research (2 , 4 , 8). Objectives Primary Objective: To identify which phase of endodontic treatment is perceived as the most difficult by students during their first clinical experience. Secondary Objectives: To determine which group of teeth is most frequently treated by undergraduate students. To assess students' self-reported confidence levels at each stage of the endodontic procedure. To quantify confidence levels specifically in the phase identified as the most challenging. To evaluate whether the perceived difficulty of the case, based on the American Association of Endodontists (AAE) classification, correlates with confidence levels in the most difficult phase. To explore whether confidence in the obturation phase is associated with the type of obturation technique used. To assess students' confidence in performing a second endodontic treatment. To identify potential improvements in endodontic education that could enhance students' confidence prior to their first clinical treatment.

## Material and Methods

This study was approved by the Research Committee of the School of Doctoral Studies and Research on 22 September 2023 (Approval Code: CI-2023-284). This cross-sectional observational study aimed to assess the perceived difficulty of endodontic procedures among undergraduate dental students. The study design, implementation, and reporting adhered to the STrengthening the Reporting of OBservational studies in Epidemiology (STROBE) guidelines, ensuring methodological rigor and transparency throughout the research process. - Study Population and Sample The study population consisted of fifth-year dental students enrolled in clinical practice who were performing their first endodontic treatment on patients. The sample size corresponded to the total number of students participating in the clinical sessions during the study period. All participants provided written informed consent prior to inclusion in the study. Inclusion criteria: Fifth-year dental students enrolled in the clinical practice course who performed their first endodontic treatment on a patient during the study period and provided informed consent. Exclusion criteria: Students repeating the year, those with prior clinical experience in endodontics, or previous dental hygiene training were excluded to ensure homogeneity of the sample. - Data Collection Instrument Data were collected using a structured questionnaire comprising three sections (Table 1):

Table 1

1. Clinical case characteristics 2. Self-reported confidence levels at each stage of the endodontic procedure 3. Open-ended questions for qualitative feedback Questions about the students' sex and age were also included. The questionnaire was specifically developed for this study. Case difficulty was classified according to the American Association of Endodontists (AAE) criteria and assigned by the supervising instructor prior to treatment. All clinical procedures were performed under continuous supervision by faculty members. Instructors monitored each step to ensure patient safety while encouraging students to carry out all stages of the treatment themselves whenever feasible. The questionnaire was administered immediately after the students completed their first root canal treatment under clinical supervision. Data collection took place between October 2023 and April 2024. - Statistical Analysis Statistical analyses were conducted using SPSS software, version 29.0. Chi-square tests were employed to explore associations between categorical variables, particularly focusing on the relationship between case difficulty (as classified by the American Association of Endodontists, AAE) and key procedural variables such as working length determination and canal obturation. For each analysis, the null hypothesis (H0) posited no significant association between the variables, while the alternative hypothesis (H0) suggested a statistically significant relationship. A p-value &lt; 0.05 was considered indicative of statistical significance. Due to the presence of contingency table cells with expected frequencies below 5, the likelihood ratio was used as a complementary measure to strengthen the interpretation of results. This approach mitigates the limitations of the Chi-square test in small sample conditions, thereby enhancing the robustness of the findings.

## Results

**Figure 1:**
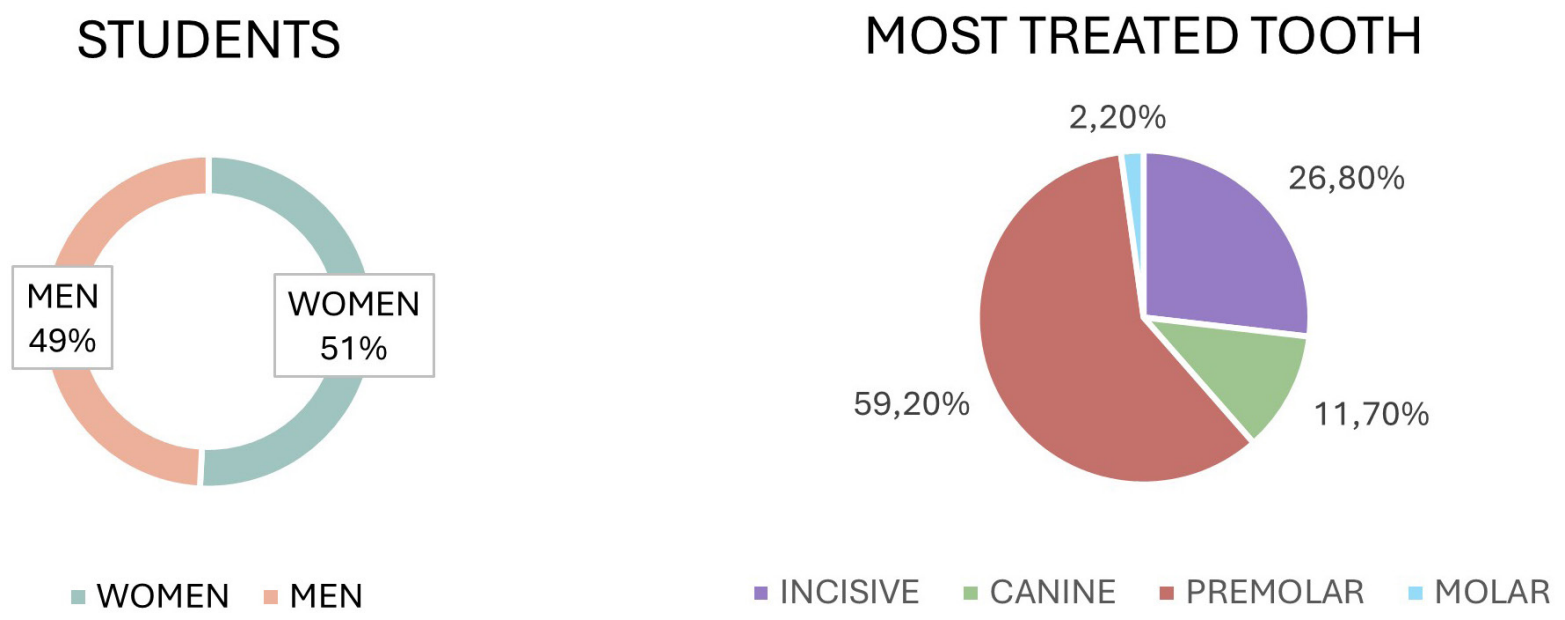
Gender of the students who completed the questionnaire represented in graph/ Percentage of the type of teeth treated by the students in graph.

a. Descriptive Analysis of Clinical Case Characteristics A total of 179 out of 200 eligible students completed the questionnaire (response rate: 89.5%). The initial cohort consisted of students enrolled in the course; however, 21 students performed their first endodontic treatment in other rotations where the research team could not administer the survey, which explains the difference between enrolment and responses. No students were excluded for other reasons. The sample included 91 female and 88 male students, with a mean age of 26.4 years. The most frequently treated tooth groups are illustrated in Figure 1.

1

Premolars were the most frequently treated teeth (59.2%; n=106), followed by incisors (26.8%; n=48). Molars accounted for 2.2% (n=4), reflecting institutional protocols by which complex molar cases are referred to the postgraduate program to prioritize patient safety and progressive competency-based exposure. Regarding case complexity, using the American Association of Endodontists (AAE) classification, 46.9% of cases were low difficulty, 50.3% moderate difficulty, and 2.8% high difficulty (the latter typically referred to the postgraduate program). Regarding obturation technique, 24.4% of students used lateral condensation and 75.6% used vertical compaction. b. Analysis of the Most Challenging Phase of Treatment Students most frequently identified canal obturation as the most difficult step (20.8%), followed by working length determination (18.0%). Canal location and instrumentation each accounted for 16.3%, and radiographic procedures 12.4%. These proportions indicate that perceived difficulty spans several stages rather than concentrating on a single phase (Table 2).

Table 2

c. Self reported confidence (by phase) Confidence levels for each phase of treatment are presented in Table 3.

Table 3

Confidence was lowest in technically demanding steps requiring precise spatial judgement and motor control: Working length determination: 26.3% reported low or very low confidence (n=47; 24.6% low, 1.7% very low). Canal obturation: 29.3% low or very low (n=52; 28.2% low, 1.1% very low). Radiographic procedures: 25.2% low or very low (n=45; 23.5% low, 1.7% very low). Management of complications: 27.9% reported low confidence. Conversely, tasks governed by standardized protocols were associated with higher confidence (e.g., diagnosis: 4.6% low or very low; local anesthesia: 2.3% low or very low). Regarding obturation technique, the survey included a question to assess whether the type of obturation (lateral vs. vertical) influenced perceived confidence There was no statistically significant association between obturation technique (lateral vs. vertical) and students' confidence (p=0.170). d. Relationship Between Case Difficulty (AAE) and Confidence in Critical Phases The distribution of case difficulty was as follows: Low difficulty: 46.9% Moderate difficulty: 50.3% High difficulty: 2.8% We tested whether AAE case difficulty influenced confidence in two critical phases (working length determination and obturation). Chi-square tests were conducted at a 95% confidence level ( = 0.05). The results were: 1. Working length determination: p-value = 0.381 (no significant association). 2. Canal obturation: p-value = 0.190 (no significant association). Thus, case complexity did not measurably impact confidence in these phases, suggesting that training factors (e.g., preclinical practice and instructor supervision) may be more influential than case difficulty per se. e. Confidence in Performing a Second Root Canal Treatment When asked about their confidence to perform a second root canal, 5.6% reported very high confidence, 28.1% high, 54.5% normal, 9.0% low, and 2.8% very low, indicating a generally positive shift in self efficacy following the first clinical exposure. Students' responses are summarized in Table 4.

Table 4

## Discussion

Endodontic treatment represents a pivotal milestone in the clinical training of dental students, particularly during their first experience treating real patients Beyond theoretical knowledge, success requires precise spatial interpretation (radiographs and apex locators), fine motor skills, and stage wise integration of techniques under supervision; factors known to elevate stress and reduce confidence during early clinical practice (2 , 3). The transition from simulated environments to clinical practice introduces unique challenges, including limited direct vision, anatomical variability of root canals, and the pressure to apply theoretical knowledge in real-time (3 , 6 , 12). Our findings align with other studies, and reflect this transition: confidence was lower in work length determination, canal obturation, and radiographic procedures, whereas protocol driven tasks (diagnosis, anesthesia) elicited higher confidence (2 , 12 , 13). This approach not only enhances learning outcomes but also helps reduce anxiety associated with complex clinical procedures. - Clinical exposure and case assignment: According to the American Association of Endodontists (AAE) and the German Endodontic Treatment Index (GETI), molars are considered the most challenging teeth due to their multiple canals, curved anatomy, and limited accessibility (14). The distribution of teeth treated (predominantly premolars and incisors, with few molars) is consistent with competency based progression and safety oriented referral patterns to postgraduate clinics. In our study, only 2.2% of cases involved molars, as institutional protocols typically refer these cases to the postgraduate endodontics program. This practice aligns with international standards that prioritize patient safety and promote a gradual, competency-based learning progression (15). In contrast, premolars (59.2%) and incisors (26.8%) were the most frequently treated tooth groups. Comparable trends have been reported in New Zealand, where maxillary premolars commonly represent the first tooth treated and clinical exposure increases across years (mean canals completed: 2.6 in fourth year vs. 10.4 in fifth year) (15). However, it is important to note that the distribution of treated teeth may reflect institutional case assignment policies rather than the students' readiness or preferences. Confidence in critical phases and alignment with prior literature: Our results identified canal obturation (20.8%) and working length determination (18%) as the most challenging phases of treatment (Table 2). Working length determination. In our cohort, 26.3% reported low/very low confidence. This aligns with reports emphasizing WL as a technically demanding step, with lower confidence or competence particularly in earlier years or with posterior teeth. For instance, Cardiff students reported limited self perceived competence-49% not competent for anterior single rooted teeth and 74% for posterior multirooted teeth-citing insufficient clinical experience as a primary reason (16). Canal obturation. Low confidence (29.3%) in obturation is consistent with prior observations that evaluation of obturation quality and radiology are among the domains with reduced confidence in undergraduates (2). The complexity of obturation is often compounded by earlier procedural shortcomings, such as insufficient canal preparation or disinfection, which can compromise apical sealing. This is supported by Sjögren et al. (1990), who highlighted the critical role of these factors in endodontic success (10). Root canal location (16.3%) also emerged as a challenging step, likely due to the steep learning curve associated with using apex locators and interpreting periapical radiographs. Awooda et al. (2016) similarly reported that the lack of immediate feedback in clinical settings contributes to student uncertainty during this phase (8). Tasks benefiting from standardized protocols. Higher confidence in diagnosis and anesthesia mirrors findings that protocol guided or repetitive tasks are less anxiety inducing than phases requiring nuanced spatial judgement and dexterity. This pattern has been noted in multi institution surveys and cohort reports (15 , 16). - Educational implications. In line with student feedback and prior recommendations, strengthening preclinical training (extracted teeth, 3D printed replicas, and simulation/VR), optimizing radiographic technique (e.g., consistent use of positioners under rubber dam), and expanding clinical exposure are likely to enhance confidence, especially in work lenght determination and obturation. Evidence from New Zealand and the UK suggests that increased case numbers and staged exposure correlate with higher confidence, particularly by the final year. Feedback from students and literature support several strategies to enhance endodontic training (5 , 8 , 16 , 17). In addition to improving obturation skills, targeted interventions for working length determination should be implemented. These may include structured workshops on apex locator calibration and radiographic verification, practical exercises using extracted teeth and anatomical replicas, and error-analysis sessions to reinforce accuracy in apical measurement. Such strategies are expected to reduce uncertainty and enhance confidence in this critical phase, ultimately improving treatment outcomes. Case complexity (AAE) did not significantly influence confidence in WL or obturation (p = 0.381 and p = 0.190, respectively), underscoring that training variables may be more decisive than anatomical difficulty in shaping students' early clinical confidence. Our data supports curricular adjustments targeting the phases with the largest confidence deficits (work length determination and obturation) and the technical enablers (radiographic positioning, apex locator proficiency) that underpin them. - Study Limitations. While this study provides valuable insights, several limitations should be acknowledged: The absence of magnification tools (e.g., dental operating microscopes) may have limited students' accuracy during critical phases such as canal location. Future studies should consider this variable. The systematic referral of complex cases to postgraduate programs limits students' exposure to anatomical variability, potentially biasing their perception of procedural difficulty.

## Conclusions

This study demonstrates that dental students perceive working length determination and canal obturation as the most challenging phases of their first endodontic treatment, regardless of the obturation technique used. These findings are consistent with previous research, which attributes the difficulty of these stages to the precision required for three-dimensional sealing and their dependence on the quality of preceding steps, such as canal instrumentation. Although not identified as the most difficult phase, root canal location also posed a significant challenge-likely due to the absence of immediate feedback in clinical settings. Students emphasized the value of enhanced preclinical training, including the use of extracted teeth, 3D-printed anatomical replicas, and virtual reality simulators, to build confidence before treating patients. These suggestions align with the European Society of Endodontology's recommendations for competency-based education. Following their first clinical experience, students reported increased confidence in performing a second endodontic treatment. This highlights the importance of expanding clinical exposure beyond the current limit of 1-2 cases per student, moving toward the 20-case benchmark recommended for achieving core clinical competence. Overall, these results underscore the need to integrate emerging technologies, enhance preclinical simulation, and increase clinical opportunities, with a particular focus on the most technically demanding phases. Such improvements are essential to support a smoother transition from supervised learning to autonomous clinical practice.

## Figures and Tables

**Table 1:** Structure and content of the questionnaire administered to students.

Questions about the case	
Type of tooth treated	Incisor - Canine - Premolar - Molar
Difficulty of the case according to the AAE	Low - Moderate - High
Most difficult phase during treatment	Diagnosis - Anaesthesia - Isolation - Access cavity - Locating canals - Working length - Instrumentation - Filling - Taking X-rays - Post-treatment instructions
Type of filling used in endodontics	Lateral - Vertical
Level of confidence in each treatment step (score 1-5 with 1 being the least confidence and 5 being the most confidence)	
Diagnosis	1-2-3-4-5
Administration of anaesthesia	1-2-3-4-5
Isolation	1-2-3-4-5
Preparation of the access cavity	1-2-3-4-5
Location of ducts	1-2-3-4-5
Determination of working length	1-2-3-4-5
Manual duct instrumentation	1-2-3-4-5
Rotary duct instrumentation	1-2-3-4-5
Duct sealing	1-2-3-4-5
Complications in treatment	1-2-3-4-5
Taking X-rays	1-2-3-4-5
Interpretation of radiographs	1-2-3-4-5
Placement of temporary restoration	1-2-3-4-5
Explanation of post-treatment instructions to the patient	1-2-3-4-5
Confidence level at which you are confident to perform the second endodontic treatment	1-2-3-4-5
Open questions	
What improvements would you include in your pre-operative endodontic training, i.e. before your first endodontic treatment?	
Suggestions for improving endodontic treatment performed by students.	

1

**Table 2:** Distribution of student responses to the question: “Which phase of the treatment was the most difficult for you?”.

Diagnosis	2.8%
Anaesthesia	0.6%
Isolation	3.4%
Access cavity	9%
Location of ducts	16.3%
Working length	18%
Instrumentation	16.3%
Sealing	20.8%
Taking X-rays	12.4%
Post-treatment instructions	0.4%

2

**Table 3:** Self-Reported confidence levels of students at each stage of endodontic treatment.

STAGE OF THE TREATMENT	VERY HIGH	HIGH	NORMAL	LOW	VERY LOW
Diagnosis	6.8%	33.3%	55.4%	4%	0.6%
Anaesthesia	29.6%	47.5%	20.7%	0,6%	1.7%
Isolation	26.8%	45.3%	24.6%	3,4%	0%
Access cavity	7.3%	32.4%	51.4%	8,4%	0.6%
Location of ducts	6.7%	26.8%	45.8%	19,6%	1.1%
Working length	1.7%	19%	53.1%	24,6%	1.7%
Manual instrumentation	3.4%	25.3%	54.5%	15,7%	1.1%
Rotary instrumentation	3.4%	28.5%	52%	15,6%	0.6%
Duct sealing	3.4%	20.9%	46.3%	28,2%	1.1%
Complications	1.2%	10.1%	60.9%	24,3%	3.6%
Taking X-rays	9.5%	24%	41.3%	23,5%	1.7%
Interpretation of radiographs	9.6%	46.1%	41.6%	2,8%	0%
Temporary restoration	18.1%	49.7%	30.5%	1,1%	0.6%
Post-treatment instructions	12.9%	37.6%	42.1%	5,1%	2.2%

3

**Table 4:** Student Confidence in Performing a Second Endodontic Treatment.

Very high	5.6%
High	28.1%
Normal	54.5%
Low	9%
Very low	2.8%

4

## Data Availability

The data that support the findings of this study are available from the corresponding author upon reasonable request.

## References

[1] De Moor R, Hülsmann M, Kirkevang LL, Tanalp J, Whitworth J (2013). Undergraduate Curriculum Guidelines for Endodontology. Int Endodontic J.

[2] Alrahabi M (2017). The confidence of undergraduate dental students in Saudi Arabia in performing endodontic treatment. Eur J Dent.

[3] Iranmanesh P, Tabatabaei SA, Saatchi M, Tahani B, Binandeh ES, Khademi A (2021). Evaluation of the Perceived Confidence of Undergraduate Dental Students in Performing Endodontic Treatment. Dental Hypotheses.

[4] Rambabu T, Srikanth V, Sajjan G, Ganguru S, Gayatri C, Roja K (2018). Comparison of tentative radiographic working length with and without grid versus electronic apex locator. Contemp Clin Dent.

[5] Martins RC, Seijo MOS, Ferreira EF, Paiva SM, Ribeiro Sobrinho AP (2012). Dental students’ perceptions about the endodontic treatments performed using NiTi rotary instruments and hand stainless steel files. Braz Dent J.

[6] Roudsari MS, Namdari M, Mortazavi H, Malek-Mohammadi M, Tohidi S (2022). Psychosocial impacts, perceived stress, and learning effects during the transition from preclinical to clinical dental education: Validation and translation of a questionnaire. Dent Res J (Isfahan).

[7] Baaij A, Özok AR (2018). Method of teaching undergraduate students to perform root canal treatment: It’s influence on the quality of root fillings. Eur J Dental Education.

[8] Awooda E, Mudathir M, Mahmoud S (2016). Confidence level in performing endodontic treatment among final year undergraduate dental students from the University of Medical Science and Technology, Sudan (2014). Saudi Endod J.

[9] Mousavi SA, Farhad A, Shahnaseri S, Basiri A, Kolahdouzan E (2018). Comparative evaluation of apical constriction position in incisor and molar teeth: An in vitro study. Eur J Dent.

[10] Sjögren U, Hägglund B, Sundqvist G, Wing K (1990). Factors affecting the long-term results of endodontic treatment. Journal of Endodontics.

[11] Souza RA, Sousa YTCS, Figueiredo JAPD, Dantas JDCP, Colombo S, Pécora JD (2012). Influence of apical foramen lateral opening and file size on cemental canal instrumentation. Braz Dent J.

[12] Tanalp J, Güven EP, Oktay I (2013). Evaluation of dental students′ perception and self-confidence levels regarding endodontic treatment. Eur J Dent.

[13] Seijo MOS, Ferreira EF, Ribeiro Sobrinho AP, Paiva SM, Martins RC (2013). Learning experience in endodontics: Brazilian students’ perceptions. J Dent Educ.

[14] Saha D, Mazumdar D, Biswas M, Chanani A, Das S (2024). Comparative evaluation of efficacy in working length determination: Radiography versus Electronic apex locators using in vivo and ex vivo methods with stereomicroscope validation. Journal of Conservative Dentistry and Endodontics.

[15] Murray CM, Chandler NP (2014). Undergraduate endodontic teaching in New Zealand: Students’ experience, perceptions and self-confidence levels: Undergraduate Endodontic Teaching in New Zealand. Aust Endod J.

[16] Davey J, Bryant ST, Dummer PMH (2015). The confidence of undergraduate dental students when performing root canal treatment and their perception of the quality of endodontic education. Eur J Dental Education.

[17] (2001). Undergraduate Curriculum Guidelines for Endodontology. Int Endodontic J.

